# Fixed Versus Lottery Incentives for Promoting Engagement With a Cadence-Based Smartphone App: Randomized Crossover Trial

**DOI:** 10.2196/86189

**Published:** 2026-05-27

**Authors:** Kosuke Hayashi, Hiromitsu Imai, Ichiro Oikawa, Hirokazu Wakuda, Iori Miura, Shingo Uenohara, Asuka Kuwae, Megumi Kai, Ken'ichi Furuya, Naoto Uemura

**Affiliations:** 1 Department of Clinical Pharmacology and Therapeutics, Faculty of Medicine, Oita University, 1-1 Idaigaoka, Hasama-Machi, Yufu, 8795593, Japan, 81 975865952; 2 Department of Cardiology, Graduate School of Medical Sciences, Nagoya City University, Nagoya, Aichi, Japan; 3 Department of Medical Ethics, Faculty of Medicine, Oita University, Yufu, Japan; 4 Clinical Pharmacology Center, Oita University Hospital, Yufu, Japan; 5 Faculty of Science and Technology, Oita University, Oita, Japan

**Keywords:** nudge, financial incentives, pedometer app, moderate-to-vigorous physical activity, MVPA, smartphone app

## Abstract

In a randomized crossover trial of 20 participants, a cadence-based smartphone app using either fixed or lottery financial incentives showed no significant difference in weekly challenge completions, daily steps, or distance, although fixed incentives may be preferable for their implementation simplicity.

## Introduction

Financial incentives have been shown to promote exercise and may be an effective strategy for improving public health [1]. Unlike other gamification elements commonly embedded in physical activity apps—such as points and levels—financial incentives provide tangible, extrinsic rewards that can independently motivate behavior change. We previously developed a smartphone app that sets cadence-based walking goals and provides financial incentives upon goal completion, focusing on intensity rather than simple step counts [2]. This approach increased moderate-to-vigorous physical activity (MVPA), which is expected to improve health outcomes that step-count targets alone might not effectively achieve. The ultimate goal of this app is to demonstrate that consistent usage can help effectively manage chronic diseases such as hypertension and diabetes. Therefore, high app adherence is crucial; however, the optimal method for delivering incentives to maintain such adherence remains unclear. Prior studies comparing fixed versus lottery incentives have yielded mixed results [3,4]. Therefore, the key aim of this study was to evaluate whether fixed or lottery incentives more effectively promote engagement with our app. Analogous to “Phase 1” in drug development, this study tested the effect of these incentive structures on app engagement in a cohort of relatively healthy volunteers. The findings of this study are intended for digital health application developers who are seeking empirical data on the optimal delivery of financial incentives.

## Methods

### App Features

The details of the app’s features and design have been previously described [2]. Briefly, the app sets cadence-based goals defined as completing 1500 steps within 15 minutes. To initiate a “walking challenge,” the user presses a “start” button in the app, which triggers a 15-minute countdown. A challenge is recorded as successful if the user completes more than 1500 steps before the timer reaches zero. These goals were not personalized in this study. A financial incentive was provided for each successful challenge, with users eligible to complete up to two challenges per day (14 per wk). Two versions of the app were developed for this study: (1) a fixed-incentive version providing ¥70 (≈US $0.50) per success, and (2) a lottery-incentive version, in which each success yielded a one-in-five chance of receiving ¥350 (≈US $2.50). Both versions had the same expected value of ¥70 (≈US $0.50) per challenge.

### Study Design and Participants

This was an 11-week randomized crossover study. Participants were assigned to use one version for 4 weeks, followed by a 3-week washout; then, they were crossed over to the other version for 4 weeks. Recruitment occurred from September 2023 to January 2024 using posters distributed at the university and nearby offices.

Inclusion criteria were age 18‐60 years, ability to walk independently without aids, ability to communicate in Japanese, and having an iPhone with consent to carry it throughout the study.

Exclusion criteria included engaging in structured exercise ≥3 times per week for ≥30 minutes at moderate intensity, physician-advised exercise restrictions, blood pressure of ≥160/110 mm Hg, or investigator’s judgment of unsuitability.

### Ethical Considerations

The study complied with the Declaration of Helsinki and the Japanese Ethical Guidelines for Clinical Research. Approval was obtained from the Institutional Review Board of Oita University Hospital (B23-004), and the trial information was registered with the University Hospital Medical Information Network (UMIN000052303) prior to commencement. Written informed consent was obtained from all participants. All participant data were anonymized and stored on secure, password-protected servers and clouds with access restricted solely to the investigators. Participants received the actual financial incentives earned during the study.

### Outcomes and Statistical Analysis

The primary end point was the difference in weekly successful challenge attempts between the two versions. Secondary end points included differences in daily step counts and walking distance.

Prior studies indicate that increasing MVPA by at least 30 minutes per week generates meaningful health improvements [1]. Since each successful challenge corresponds to approximately 15 minutes of MVPA, we determined that a between-group difference of three successful challenges per week (equating to 45 min of MVPA per wk) would be clinically significant. With an SD of 5, a sample of 24 participants would provide 80% power. Accounting for a 10% dropout, a target sample size of 27 was set. Data are expressed as mean (SD) or median (range) for continuous variables and percentages for categorical variables. Hypothesis tests were 2-tailed with a significance level of .05. A mixed-model repeated-measures analysis was applied to all outcomes using the intention-to-treat (ITT) principle.

## Results

From September 2023 to March 2024, 20 participants were enrolled. This sample size provided 80% power to detect a difference of 3.3 challenges per week. Two participants dropped out during the first sequence but were included in the ITT analysis. Their baseline characteristics were as follows: 55% of participants were female, with a median age of 26 (range 19-49) years and a mean BMI of 21.5 (SD 2.2).

**Figure 1.**
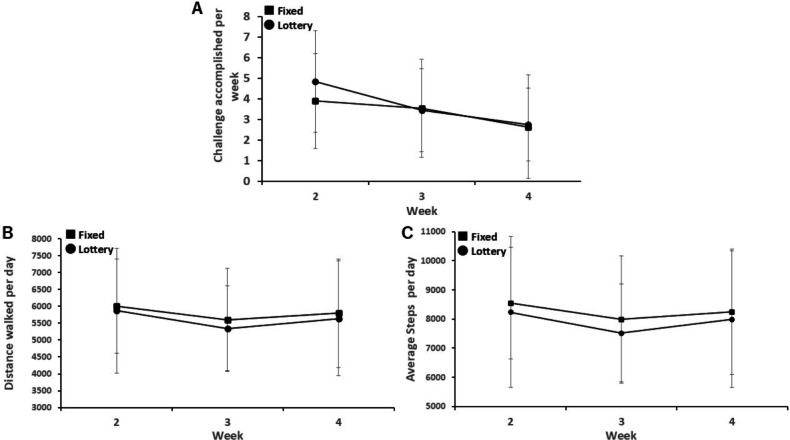
The trends of the end points over 3 weeks are shown. (A) The average number of challenges accomplished per week in the fixed and lottery incentive groups. (B) and (C)The average daily step counts and daily walking distances, respectively. Error bars represent 95% CIs.

Regarding the primary end point, there was no significant difference in successful weekly challenges between the fixed- and lottery-incentive apps (3.4 times per wk vs 3.7 times per wk; *P*=.75; Table 1 and Figure 1A). Likewise, no significant differences were observed in daily walking distance (5806 m vs 5622 m; *P*=.72; Table 1 and Figure 1B) or daily step counts (8495 steps vs 7917 steps; *P*=.59; Table 1 and Figure 1C).

**Table 1.** Outcomes.

Variable	Fixed incentive, mean (SD)	Lottery incentive, mean (SD)	*P* value
Primary end point			
Challenges accomplished per week	3.4 (4.1)	3.7 (3.4)	.75
Secondary end points			
Steps per day	8495 (3149)	7917 (3609)	.59
Distance walked per day (m)	5806 (2487)	5622 (2643)	.72

## Discussion

In this randomized crossover trial, fixed and lottery incentives produced similar levels of engagement with our app. Challenge success—our measure of engagement—requires sustained physical effort and is distinct from passive app usage. The overall challenge success rate was relatively low (3.4 to 3.7 successes per wk), suggesting that while financial incentives prompt participation, the magnitude of the reward, or the strict 15-minute time constraint, may have limited broader engagement.

Our findings contrast with those of studies highlighting the behavioral superiority of lottery models or loss-framed incentives [4], indicating that the most effective incentive structure may depend on specific app mechanics, goal difficulty, target demographics, or cultural contexts. Since neither approach showed clear superiority, we adopted the fixed-incentive model for subsequent development due to its administrative simplicity. A larger clinical trial is ongoing to evaluate whether this approach can improve long-term health outcomes.

## Abbreviations

ITT: intention-to-treat
MVPA: moderate-to-vigorous physical activity
